# Mental Health Staff Capability and Practice for Promoting Physical Activity: Evidence From a Mental Health Community‐Managed Organisation

**DOI:** 10.1002/hpja.70274

**Published:** 2026-11-02

**Authors:** Regan Lamble, Gemechu Wirtu, Andrew Watkins, Mark Orr, Jackie Curtis, Laurence Gagnon, Jade Ryall, Hamish Fibbins, Darren Haywood, Cristina M. Caperchione, David Simmons, Therese Fletcher, Nicolas H. Hart, Simon Rosenbaum, Oscar Lederman

**Affiliations:** ^1^ Human Performance Research Centre, School of Human Performance, Rehabilitation and Population Health, Faculty of Health University of Technology Sydney (UTS) Sydney New South Wales Australia; ^2^ Mindgardens Neuroscience Network Sydney New South Wales Australia; ^3^ Flourish Australia Sydney New South Wales Australia; ^4^ International Centre for Future Health Systems UNSW Medicine, University of New South Wales (UNSW) Sydney Australia; ^5^ Discipline of Psychiatry and Mental Health UNSW Medicine, UNSW Sydney Australia; ^6^ Macarthur Clinical School Western Sydney University Campbelltown New South Wales Australia; ^7^ Caring Futures Institute, College of Health and Enablement Flinders University Adelaide South Australia Australia; ^8^ Centre for Healthcare Transformation, Faculty of Health Queensland University of Technology (QUT) Brisbane Queensland Australia; ^9^ Exercise Medicine Research Institute, School of Medical and Health Sciences Edith Cowan University Perth Western Australia Australia; ^10^ Institute for Health Research University of Notre Dame Australia Perth Western Australia Australia; ^11^ Nutrition, Exercise, and Social Equity (NExuS), Discipline of Psychiatry and Mental Health Research Group, Discipline of Psychiatry and Mental Health, School of Clinical Medicine UNSW Medicine, University of New South Wales (UNSW) Sydney New South Wales Australia

**Keywords:** community managed organisation, mental health, mental health staff, physical activity, physical health, workforce capability

## Abstract

**Issue Addressed:**

People with complex mental health conditions experience substantial reductions in life expectancy, largely due to preventable chronic disease. Mental health community‐managed organisations (CMOs) are well placed to support physical health, yet little is known about staff capability to promote physical activity (PA).

**Methods:**

A cross‐sectional survey was administered to staff at a CMO in Sydney. Staff barriers, attitudes, confidence and knowledge relating to PA and physical health were assessed using the Physical Activity Attitudes Confidence Knowledge (PACK) and modified Metabolic Barriers Attitudes Confidence Knowledge (mM‐BACK) questionnaires. PA promotional practices and personal PA behaviours were also assessed and relationships between these factors explored.

**Results:**

Fifty‐six of 78 eligible staff (72%) completed the survey. Mean scores were 34.8/45 (SD = 3.6) on PACK and 43.8/65 (SD = 6.7) on mM‐BACK. Perceived barriers were generally low; workload was among those most commonly identified. Across both tools, attitudes scored highest, while confidence and knowledge scored lower. Only 23% reported familiarity with PA guidelines. Although 82% discussed PA with clients in the previous five workdays, 63% made no formal referrals. In correlation analyses, PA conversations were associated with mM‐BACK attitudes (*ρ* = 0.43, *p* < 0.001) and PACK confidence (*ρ* = 0.34, *p* = 0.011), while capability was not associated with referrals.

**Conclusions:**

CMO staff held positive attitudes towards PA promotion and commonly discussed PA with clients, but gaps remain in confidence, knowledge and referral practices.

**So What?:**

Building workforce capability, clarifying referral pathways and embedding organisational support for PA promotion within mental health CMOs may help reduce preventable health inequities in this population.

## 1 Introduction

Community‐managed organisations (CMOs) are a core part of Australia's mental health system, providing long‐term psychosocial support alongside clinical care for people with complex mental health conditions, such as schizophrenia, bipolar disorder and major depressive disorder [1, 2]. Their recovery‐oriented service models and sustained trusted relationships with the people they support (defined herein as ‘consumers’) uniquely position CMOs to influence health behaviours, including physical activity (PA). Despite this, CMOs remain overlooked in efforts to protect physical health, with an undefined role in national policy, inconsistent funding, and, in practice, resource constraints, limited training, unclear referral pathways (e.g., connecting consumers with community‐based exercise programs, structured PA initiatives or exercise professionals such as Accredited Exercise Physiologists) and ambiguity about the scope of practice [3, 4, 5].

Addressing the physical health needs of people living with complex mental health conditions is crucial, given the 10‐ to 25‐year reduction in life expectancy compared with the general population [6]. This disparity is largely attributable to higher rates of preventable cardiometabolic conditions, including cardiovascular disease, Type 2 diabetes and obesity [7, 8]. Multiple interconnected factors contribute to this gap, including systemic barriers such as limited healthcare access and fragmented care coordination; medication‐related metabolic side effects, including antipsychotic‐induced weight gain; unhealthy lifestyle behaviours, including higher rates of smoking, low nutrition quality and physical inactivity; and persistent under‐recognition of physical health needs within mental health services [9, 10, 11, 12]. As such, improving physical health outcomes is directly aligned with the CMO's role in supporting sustained, community‐based recovery.

One strategy to address these disparities is PA‐based interventions, which have been extensively investigated as an effective adjunct intervention in mental health care [7, 13]. PA is defined as people moving, acting and performing within culturally specific spaces and contexts influenced by a unique array of interests, emotions, ideas, instructions and relationships [14], while ‘exercise’ denotes planned, structured and repetitive bodily movement undertaken to improve or maintain physical fitness [15]. Regular PA is an evidence‐based strategy for preventing and managing adverse physical health outcomes (such as elevated cardiometabolic risk) that contribute to reduced life expectancy among people living with complex mental health conditions [16]. Beyond protecting and managing physical health, PA can reduce psychiatric symptoms, enhance quality of life and promote functional recovery [13]. Major policy and health bodies, including the Lancet Psychiatry Commission [17], the World Psychiatric Association [18] and Australia's Equally Well Consensus Statement [19], all identify lifestyle promotion, including PA, as central to narrowing the mortality gap. In line with these policies and guidance, integrated models involving exercise professionals have demonstrated potential to strengthen physical health care; however, their implementation remains dependent on adequate funding, clearly defined staff roles, effective referral pathways and organisational support.

Despite these well‐established benefits, a persistent implementation gap remains, with PA not translating into sustainable, system‐wide practice in routine mental health care [5, 7, 17, 20]. Barriers to implementation occur at multiple levels, including organisational constraints (workforce capacity, competing service demands, resource limitations), practitioner‐related factors (role ambiguity, limited training, low confidence) and client‐level challenges (medication side effects, low motivation) [21, 22]. Even when PA is recognised as beneficial by mental health service providers, implementation often stalls due to competing demands, limited staff confidence and a lack of PA‐trained personnel, particularly within CMOs [5, 23]. Understanding how CMO staff currently perceive and support PA is therefore important for identifying practical strategies that are feasible within CMO settings. However, evidence on staff attitudes, confidence and knowledge relating to PA promotion has largely been drawn from clinical mental health workforces and the applicability of these findings to CMOs remains unclear.

Emerging evidence suggests that CMO staff value physical health promotion but may lack the training, confidence and organisational support required to deliver it consistently. For example, a survey of NSW CMOs found that most organisations provided at least some preventive care across key behaviours, including PA, tobacco smoking, nutrition, alcohol consumption and sleep behaviour, though only 38% delivered comprehensive care across all five behaviours. Organisational enablers, such as staff training and guidelines, were inconsistently present [24]. Another study in this sector similarly found strong endorsement of physical health as part of the support worker role, alongside notable gaps in confidence, training and role clarity [25]. Mental health staff attitudes, capabilities and confidence are also key determinants of whether PA is discussed or recommended [26, 27]. However, translating this into consistent practice remains highly variable.

Despite this growing evidence base, relatively little research has examined PA promotion within CMOs. This remains a critical gap given both the central role CMOs play in routine psychosocial care and the substantial physical health burden faced by the people they support. Previous research suggests that targeted training can improve staff capability to address health behaviours, including PA, within this sector [28]. However, less is known about staff capability specifically related to physical and metabolic health, how this relates to their own PA behaviours and whether this translates into routine conversations and referral practices. To better understand this gap, this study aimed to: (1) describe mental health CMO staff capability to promote PA and physical health, defined as their attitudes, confidence, knowledge and perceived barriers; alongside their current PA promotion and referral practices and their own PA behaviours; (2) explore associations between staff capability, personal PA behaviours and the frequency of PA conversations and referrals.

## 2 Methods

### 2.1 Study Design

A cross‐sectional, observational survey design was used. Data were collected over 6 weeks, from April to May 2025. The survey was conducted online via Qualtrics XM, enabling staff to complete it anonymously via a secure web link.

### 2.2 Setting and Participants

Eligible participants were employees of Flourish Australia, a leading national mental health CMO that provides psychosocial support to people facing complex mental health challenges. Employees (referred to as 'staff’) aged 18 years or older and employed in Sydney‐based service programs were invited to participate. Only individuals directly employed by the organisation were included. Contractors, volunteers, students on placement and other non‐employees were excluded.

### 2.3 Recruitment and Data Collection

The survey was pilot‐tested with 10 eligible staff members to evaluate clarity, relevance and feasibility. Participants then completed a brief feedback form (Supporting Information Appendix A). Minor adjustments to wording and layout were made to the survey prior to distribution. Pilot test participants were not excluded from the official survey activity. All staff within select Sydney‐based program areas (*n* = 78) were invited to complete the survey via email and face‐to‐face promotion during team meetings by area managers, regardless of whether their roles involved direct support, program delivery, supervision, administration or service management.

Participant information sheets and an online consent form were provided electronically before the survey commenced. The survey was self‐administered and estimated to take approximately 10–15 min to complete. Participation was voluntary and all responses were anonymous. Ethics approval was obtained from the University of Technology Sydney (UTS) Human Research Ethics Committee (HREC ID: ETH25‐10754). Flourish Australia organisational approval was obtained prior to recruitment.

### 2.4 Outcome Measures

#### 2.4.1 Demographics

Demographic factors included age, gender, professional role, years of experience in the mental health sector and years employed at Flourish Australia.

#### 2.4.2 Staff Capability

In this study, staff capability refers to the attitudes, confidence, knowledge and perceived barriers that influence whether staff promote PA and broader physical health, as measured by the PACK and mM‐BACK questionnaires.

##### 2.4.2.1 Pack

The survey assessed staff attitudes, confidence and knowledge regarding PA promotion via the Physical Activity *Attitudes*, *Confidence* and *Knowledge* (PACK) questionnaire. The PACK is a brief, validated tool [29] designed to assess mental health professionals' perceptions and capabilities regarding PA's role in mental healthcare. PACK captures three domains to identify strengths and gaps that influence routine PA conversations, assessment and referral practices. The PACK consists of nine items across three domains: *attitudes* (3 items), *confidence* (3 items) and *knowledge* (3 items), each rated on a 5‐point Likert scale (1 = strongly disagree, 5 = strongly agree). Total scores range from 9 to 45, with each domain scored from 3 to 15. Higher scores indicate more positive attitudes, greater confidence and higher knowledge related to PA promotion. The PACK tool has demonstrated acceptable internal consistency and construct validity [29].

##### 2.4.2.2 mM‐BACK

Barriers, attitudes, confidence and knowledge regarding broader physical and metabolic health promotion were also assessed using a modified *Metabolic Barriers*, *Attitudes*, *Confidence* and *Knowledge* (M‐BACK) questionnaire. The M‐BACK is a validated tool and has been applied across clinical mental health settings, including with Australian mental health nurses [30], early psychosis staff [31] and international samples of nurses, psychiatrists and psychologists to assess staff capability to support broader physical and metabolic health [32, 33].

Given the non‐clinical nature of the CMO workforce, a modified M‐BACK (mM‐BACK) was used to assess staff perceptions of broader physical and metabolic health among those with complex mental health conditions. To ensure suitability for the CMO setting (non‐clinical), the mM‐BACK was adapted by research team members R.L., O.L. and G.W., in consultation with co‐author A.W., who developed the original M‐BACK [30]. The modifications to the M‐BACK are detailed in Supporting Information Appendix B. The *barriers* domain included four items (maximum score = 20), while the *attitudes*, *confidence* and *knowledge* domains each included three items (maximum score = 15). This adjustment reduced the maximum total score from 80 to 65. Each item was rated on a 5‐point Likert scale (1 = strongly disagree to 5 = strongly agree). Consistent with item framing (Supporting Information Appendix B), positively worded items were scored directly, while negatively worded barrier items were reverse‐coded. Higher scores, therefore, indicated more positive attitudes, greater confidence, higher knowledge and fewer perceived barriers.

#### 2.4.3 Staff PA Promotion and Referral Practices

PA promotion behaviour was assessed using two questions developed by the research team. Participants indicated (1) the number of clients with whom they had an informal conversation about PA over the past five workdays and (2) the number of formal referrals made, defined as directing or connecting a client to a specific PA program, service or professional within that timeframe. Referrals in this context could include, but were not limited to, connecting clients with community‐based exercise groups, structured PA programs or exercise professionals such as Accredited Exercise Physiologists (AEPs); referral to general practitioners for broader health matters was not the focus of this item. No threshold was applied to distinguish between higher and lower levels of promotional activity, reflecting the exploratory nature of the study and the lack of established benchmarks for PA promotion in community mental health practice.

#### 2.4.4 Personal PA Behaviours

PA levels of individual staff were assessed using the Physical Activity Vital Signs (PAVS) questionnaire. The PAVS is a brief, validated screening tool commonly used in primary care and clinical settings to assess PA levels [34]. The PAVS comprises two questions that assess the number of days per week and the average minutes per day participants engage in moderate‐to‐vigorous physical activity (MVPA), which are then multiplied to calculate total weekly MVPA. Meeting World Health Organisation (WHO) physical activity guidelines was defined as ≥ 150 min of MVPA per week, consistent with WHO recommendations [35]. The tool has been previously validated and used to assess PA levels among mental health professionals, including mental health nurses [36, 37]. In this study, the PAVS was used to assess staff PA levels and sedentary behaviour, including weekday and weekend sitting duration.

### 2.5 Statistical Analysis

Data were analysed using IBM SPSS Statistics, v29.0 (IBM Corporation, Armonk, NY). Descriptive statistics were reported as means and standard deviations for continuous variables and as frequencies and percentages for categorical variables. Internal consistency of the total scores for each tool (PACK and mM‐BACK) was assessed using Cronbach's alpha, with values of at least 0.70 considered acceptable [38]. Analyses addressing Aim 1 were descriptive, while analyses addressing Aim 2 were exploratory and hypothesis‐generating.

For Aim 1, descriptive statistics were used to characterise staff capability to promote PA and physical health, PA promotion and referral practices and personal PA behaviours. Normality of continuous variables was assessed using the Shapiro–Wilk test and inspection of Q‐Q plots.

For Aim 2, Spearman's correlations (ρ) were used to explore associations between PACK and mM‐BACK domain and total scores, personal PA behaviours (weekly MVPA and average daily sitting time) and PA promotion practices (conversations and referrals) given the non‐normal, skewed distributions of several variables. Domain scores were examined alongside total scores because attitudes, confidence, knowledge and perceived barriers are conceptually distinct components of capability that may relate differently to practice; examining them separately enabled exploration of whether particular components were more closely associated with PA conversations and referrals. These associations are presented as a correlation matrix and were interpreted as exploratory and descriptive rather than as a series of independent confirmatory hypothesis tests. No adjustment for multiple comparisons was therefore applied, with an emphasis on the pattern and magnitude of associations rather than on the significance of individual correlations.

Two exploratory forward‐selection multiple linear regression analyses were conducted, with the number of PA conversations and PA referrals as separate outcome variables. Candidate predictors for each model were the three PACK domains (attitudes, confidence and knowledge), four mM‐BACK domains (barriers, attitudes, confidence and knowledge), weekly MVPA and average daily sitting time. Forward selection was used to examine which candidate variables were retained in a smaller model for each outcome [39]. Given the modest sample size and the potential instability of data‐driven variable selection, the models and retained associations were interpreted as exploratory. Listwise deletion left 53 cases for each model. All tests were two‐tailed, with significance set at *p* < 0.05.

### 2.6 Use of Artificial Intelligence

GenAI tools (Claude, ChatGPT and Gemini) were used to support language editing and readability and all outputs were reviewed and edited by the authors, who take full responsibility for the final content.

## 3 Results

### 3.1 Participant Characteristics

The survey was distributed to 78 staff members, of whom 76 responded. After excluding incomplete surveys (*n =* 19) and duplicate entries (*n =* 1), 56 participants were included in the final analyses, yielding a completed response rate of 72%. Most participants were female (*n =* 36, 64%) and worked in community outreach programs (*n =* 32, 57%). Approximately three‐quarters (*n* = 43, 77%) had 6 years or less experience in the mental health sector (Table 1).

**TABLE 1:** Participant Characteristics (*n* = 56).

Variable		*n* (%) or M (SD)
Age (years)		M = 35.2 (SD = 11.3), range 23–63
Gender	Female	36 (64)
	Male	18 (32)
	Prefer not to say	2 (4)
	Non‐binary	0 (0)
Program area primarily worked in	Community outreach & psychosocial support	32 (57)
	Supported accommodation	17 (30)
	Other programs	7 (13)
Primary role	Mental health worker	23 (41)
	Peer worker	13 (23)
	Support & access officer	5 (9)
	Team coordinator	3 (5)
	Manager	1 (2)
	Admin/support	2 (4)
	Other	9 (16)
Professional category	Direct support	43 (77)
	Leadership	7 (13)
	Administration	2 (4)
	Other disciplines	4 (7)
Direct support role	Yes	47 (84)
	No	9 (16)
Years in the mental health sector	0–3 years	29 (52)
	4–6 years	14 (25)
	7–10 years	5 (9)
	11+ years	8 (14)^a^
Years at organisation	< 1 year	14 (25)
	1–2 years	17 (30)
	3–5 years	15 (27)
	6+ years	10 (18)^b^

*Note:* Percentages may not total 100 due to rounding.

^a^ Of those with 11 years or more sector experience (*n* = 8); two participants reported working in the mental health sector for over 21 years.

^b^ Of those employed at the organisation for 6 years or more (*n* = 10), four participants reported working at the organisation for over 10 years.

### 3.2 Internal Consistency

Across the 9 items, the PACK showed acceptable internal consistency (*α* = 0.703). For the 13‐item mM‐BACK, internal consistency was acceptable (*α* = 0.790). Internal consistency for individual PACK and mM‐BACK domains is reported in Supporting Information Appendix C. The PACK knowledge subscale (*α* = 0.223) and mM‐BACK barriers subscale (*α* = 0.224) both showed low internal consistency; given this, item‐level descriptives for these subscales are presented in Supporting Information Appendix D and the aggregated scores should be interpreted with caution.

### 3.3 Physical Activity Attitudes, Confidence and Knowledge

Nearly all respondents endorsed PA as important for mental health care; however, awareness of the WHO physical activity guidelines was limited, with only 23% reporting familiarity (Table 2). Across the PACK domains, *attitudes* towards PA promotion were rated highest (M = 12.9, SD = 1.5), followed by *confidence* (M = 11.1, SD = 1.6) and *knowledge* (M = 10.8, SD = 1.5; Table 2).

**TABLE 2:** Item‐level response frequencies and domain/scale total scores for PACK and mM‐BACK (*n* = 56).

Domain	Item	Strongly disagree *n* (%)	Disagree *n* (%)	Neutral *n* (%)	Agree *n* (%)	Strongly agree *n* (%)	M (SD)
*PACK*							
Attitudes	I believe physical activity is important for the mental health of the people I support.	0 (0)	0 (0)	1 (1.8)	27 (48.2)	28 (50.0)	4.5 (0.5)
	I believe mental health professionals should discuss physical activity as part of routine care.	0 (0)	0 (0)	4 (7.1)	26 (46.4)	26 (46.4)	4.4 (0.6)
	I see promoting physical activity as an important part of my professional practice.	0 (0)	2 (3.6)	8 (14.3)	34 (60.7)	12 (21.4)	4.0 (0.7)
	*Attitudes total*	—	—	—	—	—	12.9 (1.5)
Confidence	I am confident assessing physical activity as part of my role.	0 (0)	11 (19.6)	20 (35.7)	21 (37.5)	4 (7.1)	3.3 (0.9)
	I am confident discussing physical activity.	0 (0)	2 (3.6)	5 (8.9)	40 (71.4)	9 (16.1)	4.0 (0.6)
	I am confident linking people with available physical activity supports.	0 (0)	2 (3.6)	14 (25.0)	33 (58.9)	7 (12.5)	3.8 (0.7)
	*Confidence total*	—	—	—	—	—	11.1 (1.6)
Knowledge	I understand the relationship between physical activity and health.	0 (0)	0 (0)	1 (1.8)	31 (55.4)	24 (42.9)	4.4 (0.5)
	I know the available physical activity supports in my context.	0 (0)	5 (8.9)	14 (25.0)	31 (55.4)	6 (10.7)	3.7 (0.8)
	I know the World Health Organisation physical activity guidelines.	4 (7.1)	26 (46.4)	13 (23.2)	11 (19.6)	2 (3.6)	2.7 (1.0)
	*Knowledge total*	—	—	—	—	—	10.8 (1.5)
	PACK total score	—	—	—	—	—	34.8 (3.6)
*mM‐BACK*							
Barriers	My workload prevents me doing any health promotion activities with service users.	5 (8.9)	16 (28.6)	19 (33.9)	15 (26.8)	1 (1.8)	3.2 (1.0)
	People living with severe mental health challenges are not interested in improving their physical health.	8 (14.3)	27 (48.2)	14 (25.0)	7 (12.5)	0 (0)	3.6 (0.9)
	Informing service users about the physical health side‐effects of psychotropic medications will impact medication adherence.	3 (5.4)	14 (25.0)	23 (41.1)	14 (25.0)	2 (3.6)	3.0 (0.9)
	There is little value in supporting people with mental health challenges to improve their physical health, as the outcomes are unlikely to change.	32 (57.1)	21 (37.5)	0 (0)	3 (5.4)	0 (0)	4.5 (0.8)
	*Barriers total*	—	—	—	—	—	14.3 (2.0)
Attitudes	Facilitating and promoting physical health checks is an important part of my role.	1 (1.8)	2 (3.6)	13 (23.2)	30 (53.6)	10 (17.9)	3.8 (0.8)
	Encouraging service users to manage their nicotine use (e.g., cigarettes and vaping) is an important part of my role.	2 (3.6)	12 (21.4)	13 (23.2)	24 (42.9)	5 (8.9)	3.3 (1.0)
	Discussing nutritional intake is an important part of my role.	3 (5.4)	13 (23.2)	10 (17.9)	25 (44.6)	5 (8.9)	3.3 (1.1)
	*Attitudes total*	—	—	—	—	—	10.4 (2.6)
Confidence	I am confident in discussing health checks for metabolic syndrome with service users.	4 (7.1)	15 (26.8)	13 (23.2)	19 (33.9)	5 (8.9)	3.1 (1.1)
	I am confident in discussing smoking cessation advice to service users.	0 (0)	13 (23.2)	15 (26.8)	23 (41.1)	5 (8.9)	3.4 (0.9)
	I am confident in discussing dietary/nutritional support for metabolic syndrome for service users.	2 (3.6)	11 (19.6)	13 (23.2)	28 (50.0)	2 (3.6)	3.3 (1.0)
	*Confidence total*	—	—	—	—	—	9.8 (2.5)
Knowledge	I have a good knowledge of metabolic syndrome.	5 (8.9)	17 (30.4)	13 (23.2)	19 (33.9)	2 (3.6)	2.9 (1.1)
	I know how to organise a physical health check to screen for metabolic syndrome.	6 (10.7)	19 (33.9)	11 (19.6)	19 (33.9)	1 (1.8)	2.8 (1.1)
	I have a good understanding of the link between mental health challenges, metabolic syndrome and physical health.	1 (1.8)	6 (10.7)	13 (23.2)	33 (58.9)	3 (5.4)	3.6 (0.8)
	*Knowledge total*	—	—	—	—	—	9.3 (2.6)
	mM‐BACK total score	—	—	—	—	—	43.8 (6.7)

*Note:* Items were rated on a 5‐point Likert scale (1 = strongly disagree to 5 = strongly agree). Item‐level M (SD) reflects the 1–5 response scale; domain and scale total M (SD) reflect summed subscale/scale scores, distinct from the item‐level values above them. For mM‐BACK Barriers items, higher scores reflect fewer perceived barriers to supporting physical activity and physical health among service users due to reverse scoring. Percentages may not total 100 due to rounding.

Abbreviations: mM‐BACK, Metabolic health, Barriers, Attitudes, Confidence and Knowledge scale; PACK, Physical Activity Attitudes, Confidence and Knowledge scale.

### 3.4 Physical Health Barriers Attitudes, Confidence and Knowledge

Staff held broadly positive attitudes towards supporting consumers' physical health. However, responses were more mixed regarding some aspects of physical health promotion, with workload and concerns about medication adherence the most frequently endorsed barrier items (Table 2). Across the three‐item mM‐BACK domains, attitudes were rated highest (M = 10.4, SD = 2.6), followed by confidence (M = 9.8, SD = 2.5), then knowledge (M = 9.3, SD = 2.6). The four‐item barriers domain, scored separately due to its different item count and range, had a mean of 14.3 out of 20 (SD = 2.0), indicating relatively low endorsement of barriers after reverse scoring. At the item level, however, 28.6% of staff agreed or strongly agreed that their workload prevented them from undertaking health promotion activities (Table 2).

### 3.5 Physical Activity Promotion

Most staff (82%) reported having at least one PA conversation with consumers in the previous five workdays, with 45% having 1–2 conversations and 37% having three or more.

### 3.6 Referral Practices

Nearly two‐thirds of staff (*n* = 35, 63%) reported making no referrals in the past 5 days, while one‐third (*n* = 19, 34%) made 1–2 referrals. Only a small proportion (*n* = 2, 4%) made three or more.

### 3.7 Staff Physical Activity Vital Signs (PAVS)

On average, staff engaged in MVPA on 3.8 days per week (SD = 1.7) for approximately 49 min per day (SD = 29.6), totalling 200 min per week (SD = 167). Sedentary time averaged 6.8 h on weekdays and 5.2 h on weekends. Based on the recommendation of at least 150 min of MVPA per week, just over half of the staff (53.6%, *n* = 30) met the guideline, while 46.4% (*n* = 26) did not.

### 3.8 Exploratory Associations Between Capability, Personal PA Behaviours and PA Promotion Practices

Spearman correlations between PACK and mM‐BACK domain and total scores, personal PA behaviours and PA promotion practices are presented in Table 3. The number of PA conversations was positively associated with mM‐BACK attitudes (*ρ* = 0.43, *p* < 0.001), PACK confidence (*ρ* = 0.34, *p* = 0.011), mM‐BACK confidence (*ρ* = 0.31, *p* = 0.019) and mM‐BACK total scores (*ρ* = 0.28, *p* = 0.033). Neither capability scores, at domain or total level, nor measures of personal PA behaviour were associated with the number of PA referrals made. Both mM‐BACK and PACK total scores were negatively associated with staff sedentary time (*ρ* = −0.43, *p* = 0.001; *ρ* = −0.35, *p* = 0.010, respectively), while no clear associations were observed with weekly MVPA (*ρ* = −0.16, *p* = 0.228 for mM‐BACK; *ρ* = 0.08, *p* = 0.541 for PACK).

**TABLE 3:** Spearman correlations between capability, personal PA behaviours and PA promotion practices (*n* = 53–56).

Variable	1	2	3	4	5	6	7	8	9	10	11	12	13
1. PACK attitudes	—												
2. PACK confidence	0.27*	—											
3. PACK knowledge	0.25	0.53**	—										
4. PACK total	0.66**	0.77**	0.79**	—									
5. mM‐BACK barriers (higher = fewer barriers)	0.23	0.04	0.05	0.15	—								
6. mM‐BACK attitudes	0.22	0.45**	0.28*	0.42**	0.00	—							
7. mM‐BACK confidence	0.03	0.62**	0.35**	0.45**	0.17	0.56**	—						
8. mM‐BACK knowledge	−0.03	0.27*	0.38**	0.27*	0.12	0.31*	0.57**	—					
9. mM‐BACK total	0.16	0.52**	0.42**	0.49**	0.35**	0.71**	0.85**	0.77**	—				
10. MVPA (min/week)	0.08	0.11	−0.03	0.08	−0.11	−0.02	−0.10	−0.18	−0.16	—			
11. Sitting (hrs/day)	−0.24	−0.32*	−0.33*	−0.35**	−0.08	−0.33*	−0.36**	−0.32*	−0.43**	−0.11	—		
12. PA conversations	0.04	0.34*	0.04	0.16	0.01	0.43**	0.31*	0.07	0.28*	0.05	0.11	—	
13. PA referrals	0.11	−0.04	0.04	0.04	−0.25	0.15	0.02	−0.04	−0.02	0.06	0.14	0.37**	—

*Note:* Spearman's rho. *N* = 56 for all pairs except those involving sitting time (*N* = 53). Correlations are presented as exploratory and descriptive; no adjustment for multiple comparisons was applied.

* *p* < 0.05.

** *p* < 0.01.

### 3.9 Factors Associated With PA Conversations and Referrals

Results of the two exploratory forward‐selection regression analyses are presented in Table 4. For PA conversations, mM‐BACK attitudes were the only variable retained (*R* ^2^ = 0.178, adjusted *R* ^2^ = 0.162; *F*(1, 51) = 11.03, *p* = 0.002). Higher attitudes scores were associated with more conversations (*B* = 0.31, 95% CI [0.12, 0.50], *β* = 0.42, *p* = 0.002). For PA referrals, mM‐BACK barriers were the only variable retained (*R* ^2^ = 0.129, adjusted *R* ^2^ = 0.111; *F*(1, 51) = 7.52, *p* = 0.008; *B* = −0.17, 95% CI [−0.29, −0.05], *β* = −0.36, *p* = 0.008). Because higher barriers‐domain scores indicate fewer perceived barriers, the negative coefficient indicates fewer referrals among staff reporting fewer barriers. This unexpected association should be interpreted cautiously given the domain's low internal consistency and the absence of a corresponding rank‐based correlation with referrals. No other candidate variable met the entry criterion in either model.

**TABLE 4:** Exploratory forward‐selection multiple linear regression models for PA conversations and referrals (*n* = 53).

Model/predictor	*B*	SE	β	*t*	*p*	95% CI
PA conversations						
mM‐BACK attitudes	0.31	0.09	0.42	3.32	0.002	[0.12, 0.50]
(Constant)	−0.97	1.01		−0.96	0.340	[−2.99, 1.06]
PA referrals						
mM‐BACK barriers	−0.17	0.06	−0.36	−2.74	0.008	[−0.29, −0.05]
(Constant)	2.98	0.88		3.38	0.001	[1.21, 4.75]

*Note:* Candidate variables in each forward‐selection model were PACK attitudes, confidence and knowledge; mM‐BACK barriers, attitudes, confidence and knowledge; weekly MVPA; and average daily sitting time. The entry criterion was *p* ≤ 0.05. Only the variable shown for each outcome met that criterion. PA conversations: *F*(1, 51) = 11.03, *p* = 0.002, *R* ^2^ = 0.178. PA referrals: *F*(1, 51) = 7.52, *p* = 0.008, *R* ^2^ = 0.129. Higher mM‐BACK barriers scores indicate fewer perceived barriers.

Abbreviations: *B*, unstandardised coefficient; CI, confidence interval; SE, standard error; *β*, standardised coefficient.

## 4 Discussion

This cross‐sectional study examined mental health staff capability to promote PA and broader physical health, including attitudes, confidence, knowledge and perceived barriers, alongside their current PA promotion and referral practices and personal PA behaviours. It also explored how these factors were associated with PA conversations and referral practices. To our knowledge, this is the first study to comprehensively examine the capability to promote PA and referral practices within an Australian community‐managed mental health organisation. Staff strongly endorsed PA and physical health promotion as relevant components of mental health care. However, this positive orientation was not matched by comparable levels of confidence, knowledge or formal referral activity, suggesting a potential gap between staff willingness to support physical health and their capability to translate this into practice consistently. Exploratory analyses further suggested that different components of capability may relate differently to PA conversations and referrals.

Staff attitudes towards PA and broader health promotion, extending to support for physical health checks, nutrition discussions and addressing nicotine use, mirror findings from other mental health workforces, in which practitioners consistently recognise the importance of physical health but report substantial challenges translating this into practice.

Staff confidence in providing physical health support scored comparatively lower than their attitudes, with the lowest confidence in conducting formal assessments and conversations about metabolic health. This suggests that, although staff were generally willing to promote physical health, they may not feel equipped to do so consistently or in a structured way. Possible explanations include limited training, uncertainty about available PA supports and a lack of clearly defined PA referral options, although these factors were not directly examined in the present study. Similar findings have been reported in clinical mental health settings, where PA counselling is often provided informally because staff lack specific training and confidence [27]. Staff members' own PA behaviours may also influence how confident they feel discussing physical health and PA and whether they make referrals [40, 41]. In the present study, however, weekly MVPA showed no clear association with capability or PA promotion practices, suggesting that personal activity levels alone may not translate into greater preparedness to promote PA in CMO settings.

Knowledge represented an important capability gap across both instruments. Many staff were unfamiliar with the WHO physical activity guidelines, limiting their ability to provide evidence‐based recommendations and assess whether consumers are meeting recommended activity levels. Similarly, knowledge of metabolic syndrome and the practical steps involved in organising physical health checks was limited, suggesting that staff are being asked to support physical health in an area where they have not been adequately equipped to do so. Similar knowledge deficits have also been reported across mental health workforces more broadly and are consistent with prior research showing that although mental health staff often assume responsibility for physical health, metabolic monitoring and follow‐up are not reliably embedded in routine practice [31, 32]. Although knowledge scores were lowest, neither the PACK nor the mM‐BACK knowledge domain showed a clear exploratory association with the number of PA conversations or referrals. In contrast, attitudes and confidence were associated with conversations. The components of capability most closely linked to practice were therefore not those with the largest descriptive gaps.

Staff also expressed mixed and uncertain views about whether discussing medication side effects (e.g., weight gain) might undermine medication adherence. This uncertainty plausibly reflects gaps in medication literacy rather than negative attitudes, consistent with prior reports of non‐specialist mental health staff experiencing similar uncertainty when discussing medication‐related risks [42]. Given that most staff rejected the notion that supporting physical health has little value or that consumers are uninterested in improving their health, this uncertainty is directly relevant to PA promotion, since side‐effect‐related weight gain may provide an opportunity for raising PA with consumers. Staff who are unsure how to navigate this conversation may avoid it altogether, missing an opportunity to link medication counselling with PA support. This suggests that closing this specific knowledge gap, rather than addressing broader attitudinal barriers, may be a more targeted lever for building staff capability to promote PA in this population.

The capability gap was also evident in the contrast between frequent informal conversations and limited structured referral activity. Similar patterns have been documented across community‐managed and clinical settings, where staff endorse PA but lack the infrastructure required for consistent assessment, referral and follow‐up [25, 43]. Implementation research indicates that unclear referral processes and concerns about increased workload can reduce staff intentions to refer, even when PA programmes are available [44]. In the exploratory analyses, attitudes and confidence were associated with more frequent PA conversations, while no clear associations were observed with referrals. In the forward‐selection models, mM‐BACK attitudes were retained for PA conversations and mM‐BACK barriers were retained for referrals. Because barrier items were reverse‐scored, this coefficient indicates that staff who reported fewer barriers also reported fewer referrals, contrary to the expected direction. This association was not evident in the rank‐based correlations (Table 3). It should be interpreted with caution, given the small sample size, the low internal consistency of the barriers domain and the high proportion of staff reporting no referrals. One possibility is that staff who refer more often encounter the practical obstacles involved in doing so, which the cross‐sectional design cannot distinguish from a suppressive effect of perceived barriers on referral activity. Overall, the findings suggest that PA conversations and referrals may be influenced by various factors, including organisational factors not assessed in this study.

Staff PA levels were mixed, with approximately half meeting guideline recommendations. In the exploratory correlation analyses, weekly MVPA showed no clear associations with PA conversations or referrals. Understanding the specific occupational and structural factors that influence staff PA in CMO settings is an important direction for future research.

Staff in the present study showed a mean sedentary time of 408 min on weekdays, lower than that reported among mental health staff in clinical settings (528 min/day) [31], yet higher than general population estimates (342 min/day) [45]. Higher sedentary time was negatively associated with several capability domains across the PACK and mM‐BACK, including confidence and knowledge (Table 3). These exploratory associations suggest that sedentary behaviour may relate differently to perceived professional capability than weekly MVPA. It is possible that the capability to promote PA is influenced less by whether staff meet weekly activity targets and more by their broader engagement with movement in everyday life, which may support greater confidence and comfort when discussing PA with consumers [46].

### 4.1 Implications

These findings have several implications for strengthening PA promotion as part of broader health care support in mental health CMO settings. Given the gaps in guideline knowledge, confidence and practical skills identified in this study, there is a clear need to support staff to deliver consistent, evidence‐based physical health promotion. Tailored workforce training and educational opportunities that build practical skills, such as behaviour‐change counselling, navigating appropriate referral pathways, conducting brief PA assessments and supporting routine physical health monitoring, may help translate positive attitudes into consistent practice. These priorities are consistent with a recently proposed tiered model for CMO‐delivered physical health interventions, in which equipping staff with behaviour‐change and health‐coaching skills represents a core level of support [5].

Multi‐component workforce development programs that integrate physical health education, skills‐based workshops and simulation training have been shown to strengthen mental health staff knowledge, confidence and self‐efficacy, particularly in conducting physical health conversations and applying motivational interviewing techniques. These models demonstrate that structured, applied training builds workforce capability in community mental health contexts [47].

A further, complementary avenue is experiential, staff‐focused interventions that expose staff to the same lifestyle approaches offered to consumers. Programs such as Keeping Our Staff in Mind (KoSiM), a brief, service‐embedded intervention, have demonstrated increased staff confidence and positive changes in workplace culture, illustrating how direct engagement with physical health strategies can strengthen staff capability to promote physical health [31, 48]. In the present study, sedentary time, but not weekly MVPA, showed exploratory associations with several capability domains. Although these cross‐sectional findings do not establish that changing staff movement behaviour will improve professional capability, staff wellbeing initiatives and experiential training models may serve as complementary to traditional workforce development by building confidence, role clarity and the foundational skills needed for non‐specialist CMO staff to deliver safe, person‐centred physical and metabolic health support.

The discrepancy between positive attitudes, frequent informal conversations and limited formal referral activity highlights a potential need to strengthen referral options. Clearer referral pathways could help staff move from conversational promotion to more structured support; however, referral pathways cannot be separated from funding and service access. One option is to embed Accredited Exercise Physiologists (AEPs) within CMO services, providing more direct access to specialised PA assessment, exercise prescription and behaviour change support [5]. Where embedded AEP roles are not available, referral to external AEPs may depend on individual funding sources or access through publicly funded services. This requires strengthening partnerships with exercise professionals, simplifying referral processes and embedding options into current workflows. Evidence indicates that even modest improvements in referral clarity and feedback mechanisms can support more consistent practice [23, 49]. Importantly, because referral infrastructure was not measured directly in this study, these strategies should be considered implications for implementation rather than explanations established by the current findings.

Because PA promotion in CMOs often occurs within broad, non‐clinical roles, aligning role expectations, providing clear guidance on where PA promotion fits within existing responsibilities and demonstrating visible organisational support may help ensure that physical health promotion does not rely solely on individual efforts [25]. This aligns with evidence that workforce capability, organisational context and leadership priorities together shape whether PA promotion is sustained beyond informal, ad‐hoc practice [10].

### 4.2 Strengths and Limitations

This study has several strengths. It is among the first to examine the workforce's capability to promote PA within a CMO mental health organisation, addressing a notable gap in the literature. The 72% response rate is strong for a workforce survey. The use of two established instruments, the PACK and M‐BACK, enabled a comprehensive assessment of attitudes, confidence, knowledge and barriers. The adaptation of the M‐BACK for non‐clinical CMO roles improved contextual relevance and the inclusion of staff PA and referral data provided a richer picture of workforce capability than attitudinal measures alone.

Several limitations should be considered. The cross‐sectional design prevents causal conclusions; the observed relationship between staff movement behaviours and professional capability warrants further investigation, particularly within CMO work environments, rather than being interpreted causally. Voluntary participation may have introduced selection bias, whereby staff with a stronger interest in physical health were more likely to complete the survey, a pattern consistent with the higher proportion of staff in this sample meeting PA guidelines than the general Australian population (53.6% compared with 22.4%) [50]. Recruiting from a single community‐managed organisation in Sydney may limit generalisability to other mental health service contexts. CMOs vary considerably in size, workforce composition, service streams and organisational culture and these characteristics shift over time in response to funding and policy change. The participating organisation is among the larger CMOs nationally [24] and has an embedded AEP physical activity program, so the transferability of findings may be limited in smaller services without existing embedded physical activity programs or in CMOs operating in different geographic or funding contexts. The relatively small sample size may have reduced the ability to detect subtle effects. Reliance on self‐reported data carries a risk of recall and social desirability biases, especially for PA levels, confidence scores and referral practices.

The study also did not assess the availability of PA programs, services or exercise professionals within the geographic areas served by participating staff. Low referral rates may therefore reflect limited local referral options rather than staff capability alone and the referral findings should be interpreted with this in mind. Similarly, the item assessing awareness of the WHO physical activity guidelines may have underestimated actual staff knowledge, as respondents may be familiar with general PA recommendations without recognising them as specifically attributable to the WHO; given the substantial overlap between WHO guidelines and other widely used PA recommendations, awareness of appropriate activity levels among staff may be higher than the reported familiarity rate suggests. Additionally, the adapted M‐BACK items have not undergone formal validation within this workforce and the Barriers subscale showed low internal consistency (*α* = 0.22). This may partly reflect the small number of items within the Barriers subscale, as Cronbach's alpha can underestimate internal consistency in short scales. Overall, internal consistency for both tools was nonetheless broadly consistent with prior use among mental health staff, suggesting that the adapted instruments performed acceptably at the total‐scale level. The low internal consistency likely reflects the heterogeneity of perceived barriers in community‐managed settings, where workload pressures, beliefs about consumer motivation and concerns about treatment engagement may operate independently rather than as a single, coherent construct. Accordingly, barrier‐related findings should be interpreted with caution, placing greater emphasis on item‐level responses than on aggregated scores. Finally, the forward‐selection models were exploratory. With a modest sample, data‐driven selection may be unstable, particularly when candidate variables are correlated. The retained variables should therefore not be interpreted as definitive or as the most important factors associated with PA conversations or referrals. The approach is also data‐driven and atheoretical and does not allow variables to be retained on theoretical grounds.

### 4.3 Future Research

Future research should evaluate how capability‐building interventions can be integrated across diverse community settings and examine the roles of leadership, governance and service structures in maintaining consistent, sustainable physical health promotion. Longitudinal and intervention studies are needed to determine whether improvements in staff knowledge and confidence lead to more frequent and effective PA and physical health conversations and referral actions and to examine how organisational support influences these relationships over time. Larger samples are also needed to confirm the exploratory associations identified in this study and determine whether the factors associated with initiating PA conversations differ from those associated with progressing to formal referral. Whether capability differs across staff subgroups, including by role, tenure, program area and whether staff themselves meet PA guidelines, also warrants examination in samples large enough to support such comparisons. Future research should also examine whether occupational hazards such as desk‐based work, travel, workload and caseload demands (not directly assessed in the present study) may influence staff's PA behaviours and their capability to promote PA and physical health. Additionally, the associations between staff movement behaviours and capability observed here warrant examination using prospective designs and device‐based measures of activity. Future research would also benefit from applying an established behavioural framework, such as the Capability, Opportunity, Motivation and Behaviour (COM‐B) model [51], to examine how individual and organisational determinants interact to shape PA promotion practice in CMO settings.

This study focused specifically on PA and did not examine staff capability or practice relating to other health behaviours such as smoking, nutrition and alcohol use. This should be considered alongside previous work examining CMO staff's provision of preventive care across these broader health behaviours [24, 28], which may provide a useful basis for future studies that adopt a more comprehensive approach to physical health support. There is also a need to examine how co‐designed training approaches, developed collaboratively with CMO staff, peer workers and people with lived experience, can strengthen capability, confidence and role clarity. Co‐design may be particularly valuable in non‐specialist settings, where tailored, context‐specific training could help build shared understanding, support culturally responsive practice and more effectively embed physical health promotion within everyday workflows.

## 5 Conclusion

This study provides preliminary evidence on workforce attitudes, confidence, knowledge and perceived barriers in PA promotion practices within community‐managed mental health services. This setting has received limited empirical attention. Staff strongly endorsed physical health promotion; however, gaps in confidence, knowledge and formal referral activity indicate that positive attitudes alone are insufficient to support consistent practice. The contrast between frequent informal conversations and limited formal referrals highlights a key challenge for embedding PA within routine care.

Strengthening workforce capability will therefore require coordinated action at both individual and organisational levels. Practical training, clearer role expectations, accessible referral pathways, a PA‐promoting workplace culture and partnerships with exercise professionals represent feasible priorities for CMOs. Future multi‐site and longitudinal studies are needed to examine how workforce capability‐building and organisational supports interact to sustain PA promotion in practice. Addressing these workforce and structural factors is critical to reducing the substantial physical health inequities experienced by people living with complex mental health conditions.

## Funding

This work was supported by the Maridulu Budyari Gumal (SPHERE) ‘Big Ideas’ Health Services Research Grant Scheme (2024).

## Conflicts of Interest

M.O., L.G. and J.R. are employed by Flourish Australia, which delivers services relevant to this study. These authors contributed to the study design and interpretation; however, data collection and analysis were conducted independently. The remaining authors declare no conflicts of interest.

## Supporting information


**Appendix A.** Pilot test feedback form.
**Appendix B**. M‐BACK modification comparison.
**Appendix C**. Internal consistency (Cronbach's *α*) for PACK and mM‐BACK scales and domains.
**Appendix D**. Item‐level descriptives for the PACK Knowledge and mM‐BACK Barriers subscales (*n* = 56).

## Data Availability

The data that support the findings of this study are available on request from the corresponding author. The data are not publicly available due to privacy or ethical restrictions.
