# Supplementary material for: Mental Health Staff Capability and Practice for Promoting Physical Activity: Evidence From a Mental Health Community‐Managed Organisation
Source: Health Promot J Austr. 2026 Nov 2;38(1):e70274. doi: 10.1002/hpja.70274 (PMC13727368; doi:10.1002/hpja.70274)
Supplement: Supplementary file 1 [file HPJA-38-0-s001.docx]

## Supplementary Appendices

**Appendix A**
Pilot test feedback form

**Appendix B**
M-BACK modification comparison

|  | Original M-BACK items | Modified M-BACK items | Framing | Domain |
| --- | --- | --- | --- | --- |
| 1 | My workload prevents me from doing any health promotion activities with clients. | My workload prevents me from doing any health promotion activities with service users. | Negative | Barrier |
| 2 | Consumers with a severe mental illness are not interested in improving their physical health. | People living with severe mental health challenges are not interested in improving their physical health. | Negative | Barrier |
| 3 | Informing clients about the possible effects of medications on their physical health will increase non-adherence. | Informing service users about the physical health side-effects of psychotropic medications will impact medication adherence. | Negative | Barrier |
| 4 | Screening for metabolic syndrome and physical health interventions are pointless as poor physical health outcomes are unavoidable. | There is little value in supporting people with mental health challenges to improve their physical health, as the outcomes are unlikely to change. | Negative | Barrier |
| 5 | Metabolic health screening is an important part of my role as a mental health clinician. | Facilitating and promoting physical health checks is an important part of my role. | Positive | Attitudes |
| 6 | Giving smoking cessation advice is an important part of my role as a mental health clinician. | Encouraging service users to manage their nicotine use (e.g. cigarettes and vaping) is an important part of my role. | Positive | Attitudes |
| **-** | Encouraging consumers to increase their physical activity is an important part of my role as a mental health clinician. | **Removed due to the PACK questionnaire taking place** | Positive | Attitudes |
| 7 | Discussing nutritional intake is an important part of my role as a mental health clinician. | Discussing nutritional intake is an important part of my role. | Positive | Attitudes |
| 8 | I am confident in my ability to screen for metabolic syndrome. | I am confident in discussing health checks for metabolic syndrome with service users. | Positive | Confidence |
| 9 | I am confident in providing smoking cessation advice to consumers. | I am confident in discussing smoking cessation advice to service users. | Positive | Confidence |
| **-** | I am confident in prescribing exercise interventions to prevent/ treat metabolic syndrome. | **Removed due to the PACK questionnaire taking place** | Positive | Confidence |
| 10 | I am confident in using dietary interventions to prevent/treat metabolic syndrome in consumers. | I am confident in discussing dietary/nutritional support for service users with metabolic syndrome. | Positive | Confidence |
| 11 | I have a good knowledge of metabolic syndrome. | I have a good knowledge of metabolic syndrome. | Positive | Knowledge |
| 12 | I understand how to screen for metabolic syndrome. | I know how to organise a physical health check to screen for metabolic syndrome. | Positive | Knowledge |
| 13 | I understand how to read pathology reports for lipids and glucose results | I have a good understanding of the link between mental health challenges, metabolic syndrome and physical health. | Positive | Knowledge |
| **-** | I understand the metabolic side effects of different neuroleptic medications. | **Removed due to non-clinical focus** | Positive | Knowledge |
| *Note.* M-BACK = Metabolic Barriers, Attitudes, Confidence and Knowledge. The modified version was adapted for use in the Community in Motion staff survey to reflect contemporary terminology (e.g., service users in place of clients), non-clinical language, and alignment with the PACK (Physical activity Attitudes, Confidence and Knowledge) questionnaire. Items marked “Removed” were excluded due to duplication, overlap with PACK measures, or limited relevance to non-clinical staff roles. Framing indicates whether each item is positively or negatively worded, and “Domain” reflects the corresponding conceptual category from the original M-BACK framework. | | | | |

**Appendix C**

*Internal consistency (Cronbach’s α) for PACK and mM-BACK scales and domains*

| **Scale** | **Domain** | **Cronbach’s α** |
| --- | --- | --- |
| **PACK** | ***Total scale*** | **0.703** |
|  | Attitudes | 0.773 |
|  | Confidence | 0.497 |
|  | Knowledge | 0.223 |
| **mM-BACK** | ***Total scale*** | **0.790** |
|  | Barriers | 0.224 |
|  | Attitudes | 0.847 |
|  | Confidence | 0.754 |
|  | Knowledge | 0.813 |

Note. PACK = Physical Activity Attitudes, Confidence and Knowledge scale; mM-BACK = Metabolic health, Barriers, Attitudes, Confidence and Knowledge scale. Cronbach’s alpha values ≥ 0.70 are generally considered acceptable internal consistency; the mM-BACK Barriers subscale (α = 0.224) and PACK Knowledge subscale (α = 0.223) fell below this threshold and should be interpreted with caution.

**Appendix D**

*Item-level descriptives for the PACK Knowledge and mM-BACK Barriers subscales (n = 56)*

| **Scale** | **Item** | **M** | **SD** | **N** |
| --- | --- | --- | --- | --- |
| **PACK Knowledge** | I understand the relationship between physical activity and health. | 4.41 | 0.532 | 56 |
|  | I know the available physical activity supports in my context. | 3.68 | 0.789 | 56 |
|  | I know the World Health Organisation physical activity guidelines. | 2.66 | 0.996 | 56 |
| **mM-BACK Barriers** | My workload prevents me doing any health promotion activities with service users. | 3.16 | 0.987 | 56 |
|  | People living with a severe mental health challenges are not interested in improving their physical health. | 3.64 | 0.883 | 56 |
|  | Informing service users about the physical health side-effects of psychotropic medications will impact medication adherence. | 3.04 | 0.934 | 56 |
|  | There is little value in supporting people with mental health challenges to improve their physical health, as the outcomes are unlikely to change. | 4.46 | 0.762 | 56 |

Note. Items were rated on a 5-point Likert scale (1 = strongly disagree to 5 = strongly agree). For mM-BACK Barriers items, higher scores reflect fewer perceived barriers to supporting physical activity and physical health among service users, due to reverse scoring. Item-level descriptives are provided for the PACK Knowledge (α = 0.223) and mM-BACK Barriers (α = 0.224) subscales given their low internal consistency, which suggests these items may not represent a single unified construct within each subscale.
